# Summarizing relationships between chemicals, genes, proteins, and diseases in PubChem using analysis of their co-occurrences in patents

**DOI:** 10.1186/s13321-025-01134-w

**Published:** 2025-12-17

**Authors:** Leonid Zaslavsky, Tiejun Cheng, Asta Gindulyte, Sunghwan Kim, Paul A. Thiessen, Evan E. Bolton

**Affiliations:** National Center for Biotechnology Information, National Library of Medicine, National Institutes of Health, Bethesda, MD 20894 USA

**Keywords:** PubChem, Patent, Co-occurrence, Knowledge panel

## Abstract

The knowledge panels in PubChem allow users to quickly identify and summarize important relationships between chemicals, genes, proteins, and diseases by analyzing the co-occurrences of those entities in a collection of text documents. In the present study, the analysis and summarization techniques used to develop the literature knowledge panels in PubChem were extended to patent documents from the Google Patent Research Data (GPRD) set. The annotations of the patent documents in the GPRD set were mapped to NCBI database records to create the patent co-occurrence data. The annotations were not only from the titles and abstracts of patents but also from other parts such as claims and descriptions, greatly improving the coverage of the co-occurrence-based entity relationships in PubChem. Informativeness weights of entities were introduced in the co-occurrence and relevance score computations to account for a significant variation in the number of matched annotations per patent section. This narrows the focus to the co-occurrences that are more relevant to the subject matter of the patent. The resulting co-occurrence data was used to generate the patent knowledge panels, enabling users to identify entities co-mentioned in patents alongside a specific chemical or gene. The patent co-occurrence data can be downloaded interactively or accessed programmatically. Overall, the patent knowledge panels described in this study provide users with quick access to essential biomedical entities associated with a given PubChem record. Users can delve into relevant patent documents related to these entities or download the underlying co-occurrence data for further exploration and analysis.

**Supplementary Information:**

The online version contains supplementary material available at 10.1186/s13321-025-01134-w.

## Scientific contribution

The co-occurrence of chemicals, genes, proteins, and disease in patent documents was analyzed and summarized to develop the patent knowledge panels, which allow users to easily find entities frequently appearing with a specific chemical or gene in patent documents. The patent co-occurrence data can be downloaded, interactively or programmatically, for further analysis. The analysis methodology has been enhanced (by the authors over past efforts) to focus on the co-occurrences that are more relevant to the subject matter of the patent.

**Supplementary Information:**

The online version contains supplementary material available at 10.1186/s13321-025-01134-w.

## Introduction

PubChem (https://pubchem.ncbi.nlm.nih.gov) [1–6] is a public repository of chemicals and their biological activities. Along with other databases at the U.S. National Center for Biotechnology Information (NCBI) [7], it provides extensive resources for biological and medical discovery, serving a wide range of users, including research scientists, chemical educators, chemical hygiene officers, students, patent agents, and many others. The literature knowledge panels developed and implemented in PubChem [8] summarize important relationships between chemicals, genes, proteins, and diseases by analyzing co-occurrences of terms in biomedical literature titles and abstracts. Our co-occurrence analysis has been utilized in various projects [9, 10].

In the present study, the analysis and summarization techniques used to develop the literature knowledge panel are extended, as announced in [6], to patent documents in the “Google Patents Research Data (GPRD)”, which is based on analysis of patent data provided by IFI CLAIMS Patent Services [11] and licensed under a Creative Commons Attribution 4.0 International License. The GPRD set, containing bibliographic information on more than 130 million patents, is available on the web [12], for download using Google BigQuery [13, 14], as well as a copy from PubChem [15]. In collaboration with OntoChem [16], Google created machine annotations for the scientific content of the worldwide patent corpus which includes tens of billions of mentions of chemicals, diseases, proteins, genes, anatomy, and other entities, extracted from the full text, images, PDFs, and translations of patent documents [17–19].

While a large-scale computational analysis of information in patents could be used to explore important relationships between chemicals, genes, proteins, and diseases, the tremendous amount of patent data and the heterogeneity of annotations in the documents in the GPRD set require a novel approach to summarize relevant information accurately and reliably. Particularly, a significant variation from patent to patent in the number of entities appearing in patent document sections necessitates a rework of the scoring functions used in the previous study [8] to compute the co-occurrence scores between the query entity and its neighbors and the relevance scores for documents (i.e., patents or patent families) where the query and neighbors are co-mentioned.

Our analysis approach allows for efficient parallel processing and produces a compact and easy-to-comprehend dataset, organized around useful and informative content. The co-occurrence data from this study is used to generate the patent co-occurrence panels in chemical and gene record pages in PubChem.

## Materials and methods

### Dataset

In our analysis, we used the subset of the GPRD dataset containing granted patents from the US, European, Japanese, and Korean Patent Offices. While our previous study [8] used natural language processing (NLP) software [20] to identify named entities from PubMed records, the present study used entities annotated (found within the patent text by name recognition or analysis of figures) by Google and OntoChem [17–19]. Each annotation downloaded included a value of the proprietary confidence score. We considered annotated entities that had values of the proprietary confidence score equal to or greater than 0.4. This confidence score threshold was recommended by the Google/OntoChem team via private communication. The types of annotations used in the study are listed in Table 1. The Google/OntoChem annotations for chemicals are broken into several classes, including *chemCompound, drugs, inorgmat, natprod, polymers, substances*, and all these annotation types were mapped to compounds in PubChem. In addition, Google/OntoChem annotations of the types *humangenes* and *proteins* were mapped to gene records in PubChem. It is noteworthy that the present study does not distinguish between genes and proteins but treats them in a single category (*i.e.*, genes), because it is very difficult and often impossible to distinguish the name of a gene from the name of the protein encoded by that gene. The method used for mapping from Google/OntoChem annotations to PubChem records is described in the next section.

**Table 1:** Selection of Google/OntoChem annotation types extracted

PubChem annotation type	Google/OntoChem annotation type
Chemical	chemCompound drugs inorgmat natprod polymers substances
Disease	diseases
Gene	humangenes proteins

When analyzing entity co-occurrences in patents, we group them by *patent families*. According to the European Patent Office (EPO), “a patent family is a collection of patent applications covering the same or similar technical content” [21]. The U.S. Patent and Trademark Office (USPTO) also uses a similar definition [22, 23]. The patent family assignments provided by IFI CLAIMS with the dataset [24] were downloaded from BigQuery.

### Matching the annotated entities to the database identifiers

Chemical annotations were matched to PubChem compounds using InChIKeys [25]. Accordingly, only the annotations that have associated InChIKeys were considered. Diseases were matched to MeSH identifiers; and gene and protein annotations were matched to gene symbols, protein accession numbers, or the Enzyme Commission (EC) numbers using the procedure developed in our previous study [8]. As mentioned above, since it is difficult and often impossible to distinguish the name of a gene from the name of the corresponding encoded protein, gene and protein names are considered a single category, *gene*. Corresponding names are resolved to the most reasonable gene, protein, or enzyme symbol. As mentioned in our previous study [8], the procedure prioritizes human genes and proteins.

### Redundancy elimination

Some compounds in PubChem are very similar. That includes different salt forms of the same parent compound as well as compounds with the same parent-connectivity structure, or having the same synonym. For example, PubChem CID 5960 (L-aspartic acid) has very similar compounds CID 44367445 ((3S)-3-azaniumyl-4-hydroxy-4-oxobutanoate) and CID 139060126 (D,L-aspartic acid). If such compounds happen to be top neighbors to the query compound, the knowledge panel will be clogged with seemingly redundant information, resulting in diminished utility. This redundancy was removed by selecting a representative neighbor from each “group” of neighbors with either the same parent-connectivity group [26], or the same chemical name. We utilized the procedure described in our previous study [8].

### Scores

Keeping the terminology consistent with our previous work [8], we use *co-occurrence scores* to rank neighbor entries of a given type, and *relevance scores* to rank patent families in relation to the query-neighbor pair. However, the score functions are fundamentally reworked in comparison to the previous study [8].

**Fig. 1:**
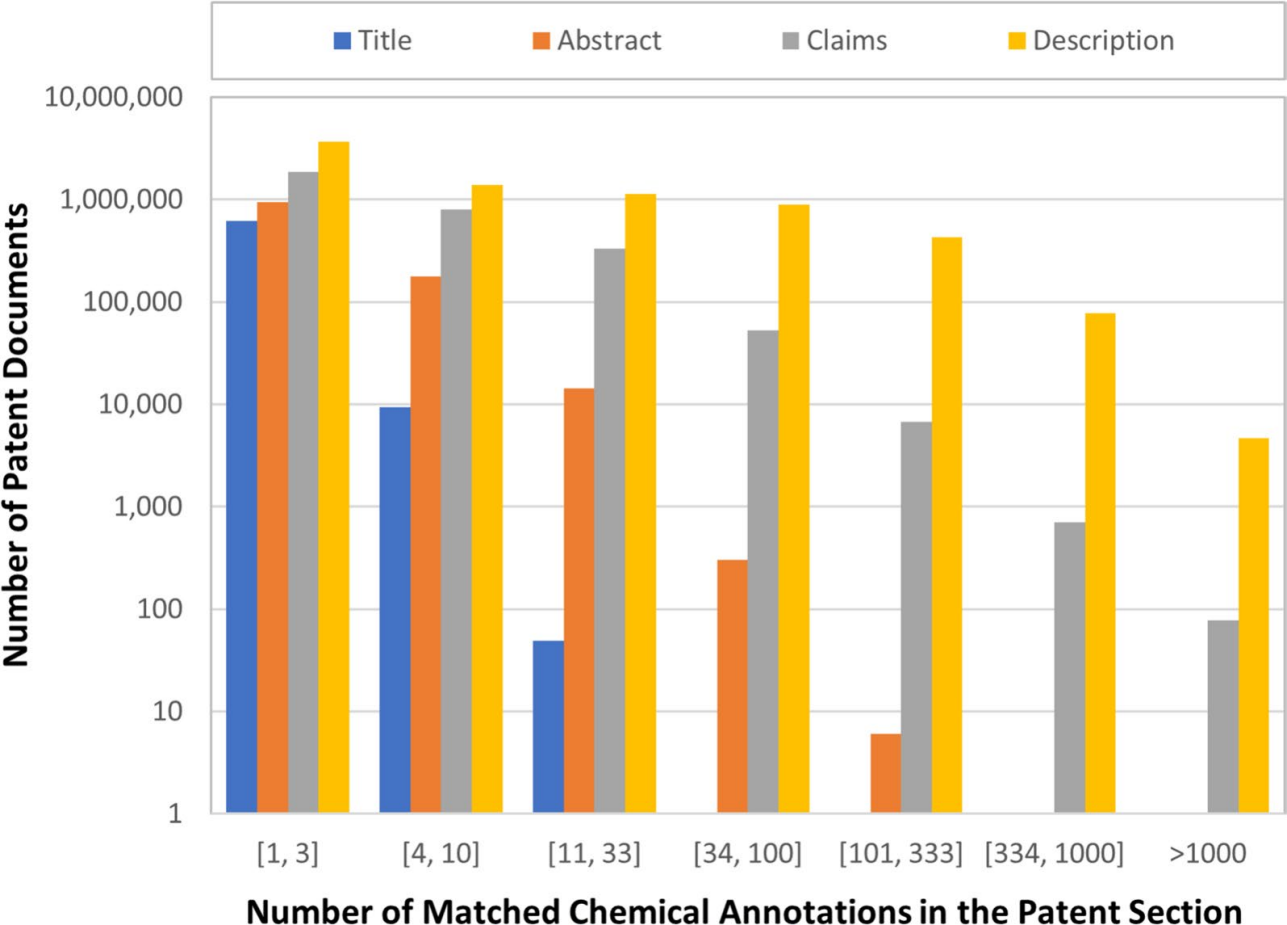
Number of matched chemical annotations in patent sections: title, abstract, claims, and description. If a chemical annotation appeared multiple times in a patent section, it was counted only once

In the co-occurrence analysis of entities annotated in PubMed records (which contain abstracts and titles) [8], all co-mentions were assumed to be indicators of some relation between the entities. We counted all co-occurrences in records, with only weighing for the frequency of the neighbor entity in the dataset. However, the situation is more complex with co-mentions of entities in patents. While the PubMed records are usually short (being just a title and abstract), and written with the intent to be scientifically clear, patents are much longer and written in a more general or vague approach, as may be necessary with legal and commercial motivations being primary considerations. There is a significant variation in the number of matched annotations per patent section (title, abstract, claims, description, etc.), with the numbers of matched chemical annotations in patent sections shown in Fig. 1. In general, the description section has the greatest number of chemicals mentioned, followed by the claims, abstract, and title sections.

A large number of annotations of the same type (usually chemicals) in the same patent section may result from the desire of the inventors and patent assignees to provide the most general descriptions possible, as well as our limited ability to extract knowledge from the patents: to distinguish mentions important to the subject matter of the patent from prior art (*e.g.*, which may give a context), routine processes, solvents, or descriptions of function and reactions, as well as to identify molecular fragments. With the current lack of deep understanding of the reasons behind each annotation, simple heuristics are needed to incorporate the annotation quality into the scoring functions. We utilize a simple model, assuming that an annotation from a patent section is useful and informative when the section contains a relatively small number of annotations of the corresponding type and that it is not useful when a patent section contains a relatively large number of annotations of a corresponding type, becoming effectively worthless for the analysis at some point.

**Fig. 2:**
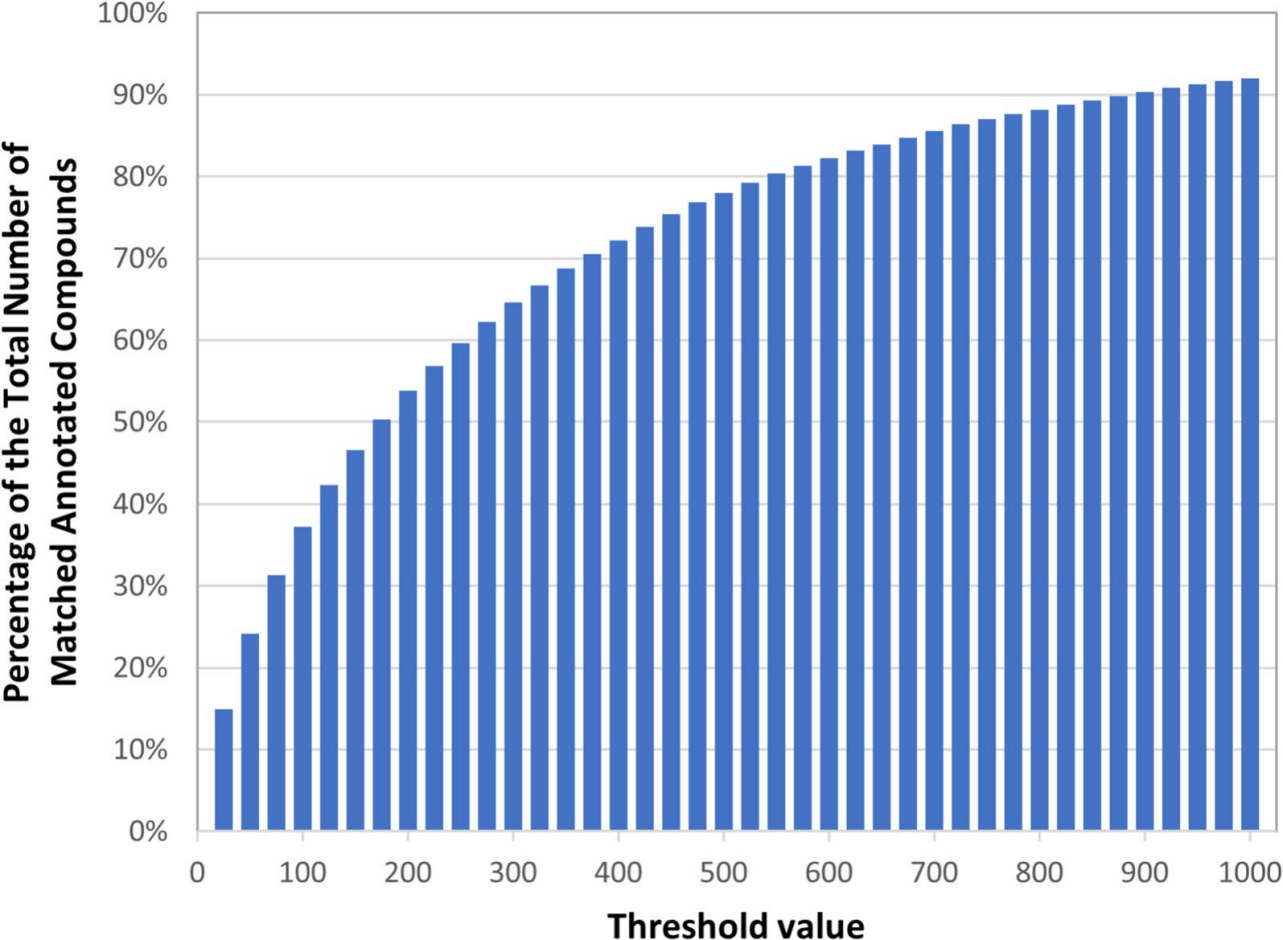
Portion of matched annotated compounds that appear in at least one patent section in which the total number of matched annotated compounds does not exceed a given threshold value (ranging from 25 to 1000 with increments of 25). For example, the first bar (at the threshold value of 25) shows that approximately 15% of the compounds are found in patent sections containing no more than 25 annotated compounds, while the remaining 85% of the compounds are found only in patent sections that mention more than 25 compounds

Figure 2 shows the portions of the matched annotated compounds that are present in the patent sections with the number of matched annotated compounds not exceeding a given threshold (for threshold values 25, 50, 75, …, 1000). Only 14.95% of matched annotated compounds appeared in at least one patent section containing 25 compounds or less. On the other hand, 92.04% of matched compounds appeared in at least one patent section containing 1000 chemicals or less, indicating that the remaining 7.96% of the matched compounds were only found in patent sections with more than 1000 matched compounds. That is, some compounds were annotated and matched only in sections with large lists of compounds. A cut-off threshold value of 200 was used (see Table 2), resulting in 53.85% of matched annotated compounds being included. This selection reflects several considerations; primarily, that we cover the majority of the compounds while limiting the count of co-occurrences within a given patent or patent family as it scales as a function of the square of the threshold (*e.g.*, 200 compounds would have 19,900 unique co-occurrence pairs). We developed an adaptive approach to gradually decrease the contribution of the annotations from patent sections with larger counts of annotations per section, before applying the cut-off threshold. For example, if the description section includes more than 200 annotated compounds, compounds mentioned within the description are excluded from consideration.

**Table 2:** Parameters used in calculations

Parameter description	Value
${N}_{s}$ for all sections except Description	5
${N}_{s}$ for section Description	20
${N}_{\text{min}}=\underset{s}{\text{min }}{N}_{s}$	5
Cut-off value ${N}_{\text{cutoff}}$	200
Max. number of non-redundant neighbors per query saved	100
Max. number of patent families per query-neighbor pair shown on the web	5
Max. number of patent families per query-neighbor pair saved for download	1000
Google/OntoChem confidence score threshold	0.4

In addition to the cut-off threshold, we define a co-occurrence score for a pair of entities in terms of co-occurrence weight for that pair within a patent family, with the co-occurrence weight being a real number between 0 and 1. It expresses the contribution of co-mentioning of the pair of entities in the patent section (patent, patent family) to the co-occurrence score of the query and neighbor entities. These co-occurrence weights are expressed in terms of informativeness weights of the individual entities which, in turn, depend on the number of entities of the corresponding type in the patent section.

### Co-occurrence scores

Let ${W}_{{i}_{1}{i}_{2}}^{({t}_{1}, {t}_{2},{s}_{1},{s}_{2},p)}$ be the co-occurrence weight for query entity ${i}_{1}$ of type ${t}_{1}$ located in the section ${s}_{1}$ of the patent $p$, and neighbor entity ${i}_{2}$ of type ${t}_{2}$ located in the section ${s}_{2}$ of the same patent (${t}_{1}\ne {t}_{2}$ or ${i}_{1}\ne {i}_{2})$:

1 $${W}_{{i}_{1}{i}_{2}}^{({t}_{1}, {t}_{2},{s}_{1},{s}_{2},p)}={W}^{({t}_{1}, {t}_{2},{s}_{1},{s}_{2},p)}{\theta }_{{i}_{1}}^{({t}_{1},{s}_{1},p)}{\theta }_{{i}_{2}}^{({t}_{2},{s}_{2},p)}$$

where ${\theta }_{i}^{(t,s,p)}$ is the Kronecker function (${\theta }_{i}^{(t,s,p)}=1$ if the entry $i$ is annotated in the section $s$ of the patent $p$, and ${\theta }_{i}^{(t,s,p)}=0$ otherwise). ${W}^{({t}_{1}, {t}_{2},{s}_{1},{s}_{2},p)}$ is defined by the empirical formula expressed in terms of informativeness weights of entities *i*_*1*_ and *i*_2_ in the next section. Type $t$ is one of three kinds: chemical, disease, and gene. Patent section $s$ of the patent may be title, abstract, claims, description, etc. Note that the value of ${W}^{({t}_{1}, {t}_{2},{s}_{1},{s}_{2},p)}$ is the same for all entities ${i}_{1}$ of type ${t}_{1}$ annotated in the section ${s}_{1}$ of the patent $p$ and all entries ${i}_{2}$ of type ${t}_{2}$ annotated in the section ${s}_{2}$ of the same patent.

The co-occurrence weight ${W}_{{i}_{1}{i}_{2}}^{({t}_{1}, {t}_{2},p)}$ for the query entity ${i}_{1}$ of type ${t}_{1}$ and the neighbor entity ${i}_{2}$ of type ${t}_{2}$ (${{t}_{1}\ne {t}_{2}\text{ or }i}_{1}\ne {i}_{2})$ in the patent $p$ is defined as:

$$W_{{i_{1} i_{2} }}^{{\left( {t_{1} , t_{2} ,p} \right)}} \, = \mathop {\max }\limits_{{s_{1} ,s_{2} }} \,W_{{i_{1} i_{2} }}^{{\left( {t_{1} , t_{2} ,s_{1} ,s_{2} ,p} \right)}}$$

and co-occurrence weight ${W}_{{i}_{1}{i}_{2}}^{({t}_{1}, {t}_{2},F)}$ for the query entity ${i}_{1}$ and the neighbor entity ${i}_{2}$ in the patent family $F$ is defined as

2 $${W}_{{i}_{1}{i}_{2}}^{({t}_{1}, {t}_{2},F)}=\underset{p\in F }{\text{max }} {W}_{{i}_{1}{i}_{2}}^{({t}_{1}, {t}_{2},p)}$$

Now we will define the co-occurrence score for a pair of entities in terms of co-occurrence weights. Let us introduce a simple co-occurrence score defined as the sum of the co-occurrence weights ${W}_{{i}_{1}{i}_{2}}^{({t}_{1}, {t}_{2},F)}$:

$${\widetilde{S}}_{{i}_{1}{i}_{2}}^{({t}_{1}, {t}_{2})}=\sum_{F}{W}_{{i}_{1}{i}_{2}}^{({t}_{1}, {t}_{2},F)}$$

between the query entity ${i}_{1}$ of type ${t}_{1}$ and the neighbor entity ${i}_{2}$ of type ${t}_{2}$ (${t}_{1}\ne {t}_{2}$ or ${i}_{1}\ne {i}_{2})$.

To take into consideration the overall frequency of the entity ${i}_{2}$ in the dataset, we introduce the inverse dataset frequency term ${r}_{{i}_{2}}^{({{t}_{1},t}_{2})},$ and define the co-occurrence score ${S}_{{i}_{1}{i}_{2}}^{({t}_{1}, {t}_{2})}$ as a product of the simple co-occurrence score ${\widetilde{S}}_{{i}_{1}{i}_{2}}^{({t}_{1}, {t}_{2})}$ and the inverse dataset frequency term ${r}_{{i}_{2}}^{({{t}_{1},t}_{2})}$:

3 $${S}_{{i}_{1}{i}_{2}}^{({t}_{1}, {t}_{2})}= {\widetilde{S}}_{{i}_{1}{i}_{2}}^{({t}_{1}, {t}_{2})} {r}_{{i}_{2}}^{({{t}_{1},t}_{2})}$$

where ${r}_{{i}_{2}}^{({{t}_{1},t}_{2})}$ is the inverse dataset frequency term for the neighbor entity ${i}_{2}$ of type ${t}_{2}$ in the type-${t}_{1}$-type-${t}_{2}$ dataset ($0\le {r}_{{i}_{2}}^{\left({{t}_{1},t}_{2}\right)}\le 1)$. This is an analogue of formula (3) in our previous paper [8] that extended the concept of the inverse database frequency (IDF) terms in classical text search algorithms [27–29] to the co-occurrence analysis. We use the empirical formula:

4 $${r}_{{i}_{2}}^{({{t}_{1},t}_{2})}=\left\{\begin{array}{c} 1-\frac{\text{log}{\overline{N} }_{{i}_{2}}^{\left({t}_{1},{t}_{2}\right)}}{\text{log}{N}_{DS}^{\left({t}_{1},{t}_{2}\right)}}, \text{ if } {N}_{DS}^{\left({t}_{1},{t}_{2}\right)}>1 \text{ and } {\overline{N} }_{{i}_{2}}^{\left({t}_{1},{t}_{2}\right)}\ge 1\\ 1, \text{ otherwise }\end{array}\right.$$

where ${N}_{DS}^{\left({t}_{1},{t}_{2}\right)}$ is the number of patent families in the type-${t}_{1}$-type-${t}_{2}$ dataset, and

5 $${\overline{N} }_{{i}_{2}}^{\left({t}_{1},{t}_{2}\right)}=\sum_{F} \underset{{i}_{1} : {i}_{1} : {t}_{1}\ne {t}_{2} \vee {i}_{1}\ne {i}_{2}}{\text{max}}{W}_{{i}_{1}{i}_{2}}^{({t}_{1}, {t}_{2},F)}$$

Let us explain the origin of the term $1-\frac{\text{log}{\overline{N} }_{{i}_{2}}^{\left({t}_{1},{t}_{2}\right)}}{\text{log}{N}_{DS}^{\left({t}_{1},{t}_{2}\right)}}$. The standard IDF term would look like $\text{log}\left(\frac{{N}_{DS}^{\left({t}_{1},{t}_{2}\right)}}{{\overline{N} }_{{i}_{2}}^{\left({t}_{1},{t}_{2}\right)}}\right)$. Since $\text{log}\left(\frac{{N}_{DS}^{\left({t}_{1},\right)}}{{\overline{N} }_{{i}_{2}}^{\left({t}_{1},{t}_{2}\right)}}\right)=\text{log}{N}_{DS}^{\left({t}_{1},{t}_{2}\right)}\left(1-\frac{\text{log}{\overline{N} }_{{i}_{2}}^{\left({t}_{1},{t}_{2}\right)}}{\text{log}{N}_{DS}^{\left({t}_{1},{t}_{2}\right)}}\right)$, we used normalization by $\text{log}{N}_{DS}^{\left({t}_{1},{t}_{2}\right)}$ to come to (4), in the same way as in formula (3) in [8].

To further explain formula (4), let us consider the properties of the term $1-\frac{\text{log}{\overline{N} }_{{i}_{2}}^{\left({t}_{1},{t}_{2}\right)}}{\text{log}{N}_{DS}^{\left({t}_{1},{t}_{2}\right)}}$:

- If ${N}_{DS}^{\left({t}_{1},{t}_{2}\right)}=1$, then $\text{log}{N}_{DS}^{\left({t}_{1},{t}_{2}\right)}=0$, and the term $\frac{\text{log}{\overline{N} }_{{i}_{2}}^{\left({t}_{1},{t}_{2}\right)}}{\text{log}{N}_{DS}^{\left({t}_{1},{t}_{2}\right)}}$ is not defined. In this case, ${r}_{{i}_{2}}^{({{t}_{1},t}_{2})}$ is set to 1(The situation when ${N}_{DS}^{\left({t}_{1},{t}_{2}\right)}=1$ does not have any practical interest, of course).
- In all practical cases, ${N}_{DS}^{\left({t}_{1},{t}_{2}\right)}>1$. Since ${N}_{DS}^{\left({t}_{1},{t}_{2}\right)}\ge$ ${\overline{N} }_{{i}_{2}}^{\left({t}_{1},{t}_{2}\right)}$ for any ${i}_{2}$, it is guaranteed that $1-\frac{\text{log}{\overline{N} }_{{i}_{2}}^{\left({t}_{1},{t}_{2}\right)}}{\text{log}{N}_{DS}^{\left({t}_{1},{t}_{2}\right)}}\ge 0$.
- However, it is possible that $0<{\overline{N} }_{{i}_{2}}^{\left({t}_{1},{t}_{2}\right)}<1$. Then $\text{log}{\overline{N} }_{{i}_{2}}^{\left({t}_{1},{t}_{2}\right)}<0$ and $1-\frac{\text{log}{\overline{N} }_{{i}_{2}}^{\left({t}_{1},{t}_{2}\right)}}{\text{log}{N}_{DS}^{\left({t}_{1},{t}_{2}\right)}}>1$. Formula (4) sets ${r}_{{i}_{2}}^{({{t}_{1},t}_{2})}$ to 1 in cases like this.

Formula (4) provides a generalization of the formula (3) from [8] (Formula (4) is reduced to formula (3) from [8] if all values of ${W}_{{i}_{1}{i}_{2}}^{({t}_{1}, {t}_{2},F)}$ are equal to 1).

### Defining informativeness weights and co-occurrence weights

**Fig. 3:**
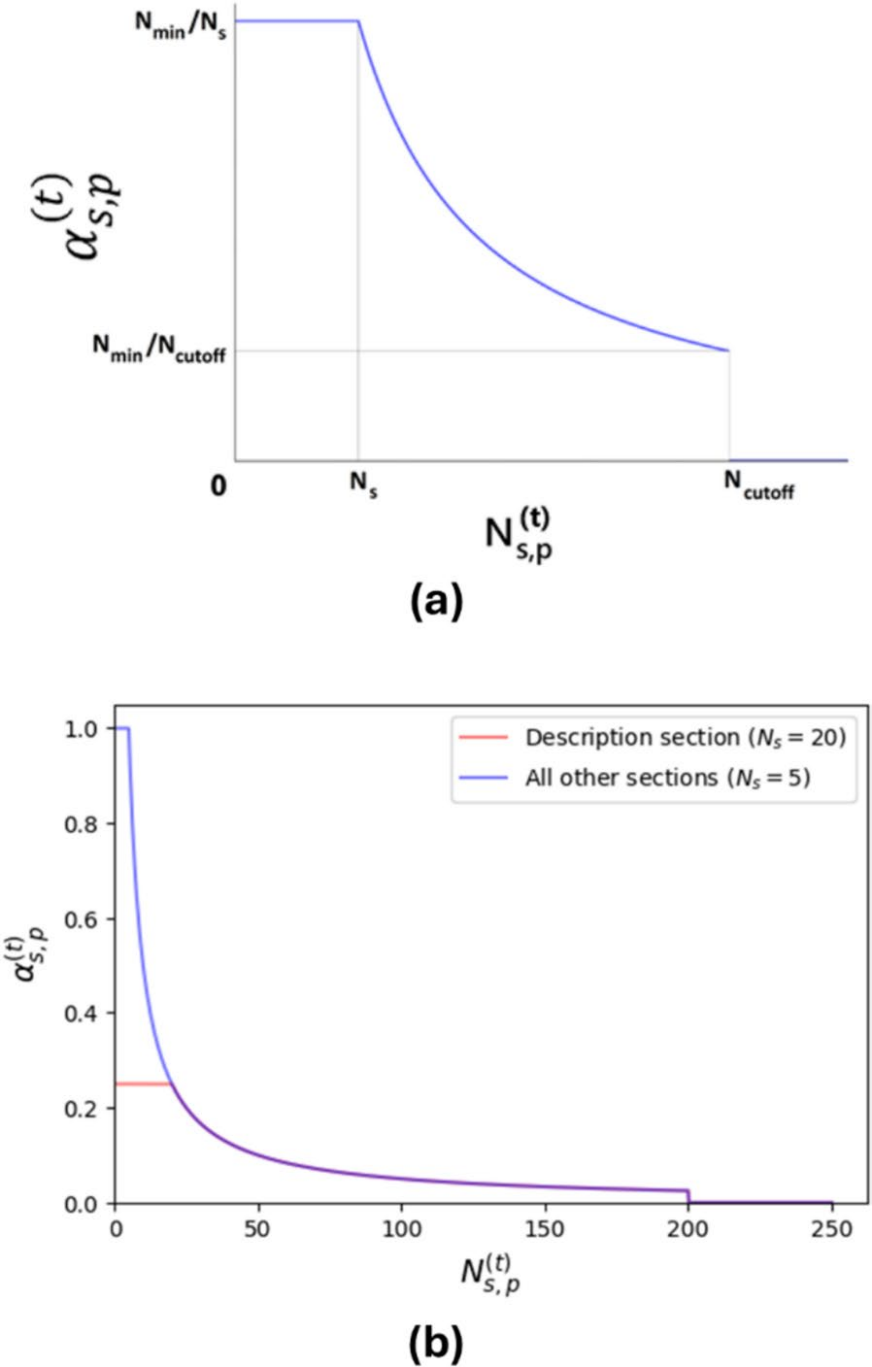
Informativeness weight ${\alpha }_{s,p}^{\left(t\right)}$ for entities of type $t$ in the section $s$ of patent *p*. **a** Illustration of the formula (6). **b** Curves for the ${\alpha }_{s,p}^{\left(t\right)}$ with the parameter values provided in Table 2

The informativeness weight ${\alpha }_{s,p}^{\left(t\right)}$ is a number between 0 and 1 having the same value for all entities of type $t$ annotated in the section $s$ of the patent $p$. It is used to gradually decrease the contribution of the matched annotations from patent sections with larger counts of matched annotations per section, before applying a cut-off. The following empirical formula for the informativeness weight is utilized:

6 $${\alpha }_{s,p}^{\left(t\right)}=\left\{\begin{array}{c}{N}_{\text{min}}/{N}_{s}, \text{ if } 0\le {N}_{s,p}^{\left(t\right)}\le {N}_{s} \\ {N}_{\text{min}}/\text{max}\left({N}_{s},{N}_{s,p}^{\left(t\right)}\right), \text{ if } {N}_{s}\le {N}_{s,p}^{\left(t\right)}\le {N}_{\text{cutoff}} \\ 0, \text{ if } {N}_{\text{cutoff}}< {N}_{s,p}^{\left(t\right)} \end{array}\right.$$

where $t$ is the entity type, $s$ is the patent section, $p$ is the patent, ${N}_{s,p}^{\left(t\right)}$ is the number of non-redundant entities of type $t$ annotated in the section $s$ of the patent $p$, $\left\{{N}_{s}\right\}$ are integer constants defined for each patent section, ${N}_{\text{cutoff}}$ is the cut-off threshold, and ${N}_{\text{min}}=\underset{s}{\text{min }}{N}_{s}$. [Note that, for the non-redundant entities, one representative from each group of near-redundant chemical entities (described in the section “Redundancy Elimination”) is counted. For other types, each entity is counted.] The formula (6) is illustrated in Fig. 3(a). The values of ${N}_{s}$ are empirically determined and listed in Table 2. The curves for ${\alpha }_{s,p}^{\left(t\right)}$ parameter values are shown in Fig. 3(b).

We define the term ${W}^{({t}_{1}, {t}_{2},{s}_{1},{s}_{2},p)}$ in Eq. (1) as a product of ${\alpha }_{{s}_{1},p}^{\left({t}_{1}\right)}$ and ${\alpha }_{{s}_{2},p}^{\left({t}_{2}\right)}$:

7 $${W}^{({t}_{1}, {t}_{2},{s}_{1},{s}_{2},p)}={\alpha }_{{s}_{1},p}^{\left({t}_{1}\right)}{\alpha }_{{s}_{2},p}^{\left({t}_{2}\right)}$$

where ${\alpha }_{{s}_{1},p}^{\left({t}_{1}\right)}$ is the informativeness weight for query entity ${i}_{1}$ of type ${t}_{1}$ located in the section ${s}_{1}$ of the patent $p$ and ${\alpha }_{{s}_{2},p}^{\left({t}_{2}\right)}$ is the informativeness weight for the neighbor entity ${i}_{2}$ of type ${t}_{2}$ located in the section ${s}_{2}$ of the same patent.

### Relevance scores

Relevance scores are used for ordering patent families in relation to the co-occurrence of the query and neighbor entities. We denote the relevance score for the patent family $F$ the co-occurrence of the query entity ${i}_{1}$ of type ${t}_{1}$ and neighbor entity ${i}_{2}$ of type ${t}_{2}$ (${t}_{1}\ne {t}_{2}$ or ${i}_{1}\ne {i}_{2})$ as ${R}_{{i}_{1}{i}_{2}}^{({t}_{1}, {t}_{2},F)}$. In this study, we do not explicitly consider the closeness of the annotation locations in the text. As a result, the relevance score ${R}_{{i}_{1}{i}_{2}}^{({t}_{1}, {t}_{2},F)}$ could be simply set equal to the co-occurrence weight ${W}_{{i}_{1}{i}_{2}}^{({t}_{1}, {t}_{2},F)}$ introduced in formula (2) above:

8 $${R}_{{i}_{1}{i}_{2}}^{({t}_{1}, {t}_{2},F)}={W}_{{i}_{1}{i}_{2}}^{({t}_{1}, {t}_{2},F)}$$

If, however, the relevance score would have explicitly accounted for the closeness of the annotation locations in the text, such as in [8], ${R}_{{i}_{1}{i}_{2}}^{({t}_{1}, {t}_{2},F)}$ and ${W}_{{i}_{1}{i}_{2}}^{({t}_{1}, {t}_{2},F)}$ would differ.

### An exclusion list for chemical compounds

When chemicals are mentioned in a patent, they are not always the subject matter of the patent. They can be in relation to production processes, as a solvent (or some other common chemical), the function of the novel molecule and reactions, or to describe a chemical fragment that is part of a Markush structure definition. Typically, applying an inverse dataset frequency term is not enough to downgrade such chemicals in the neighbor list. To make the neighbor list more informative, we created an exclusion list using the following two criteria:

- We selected a list of 250 compounds with the largest counts of annotated patent families across the entire dataset.
- We selected 150 compounds with the largest counts of patent families that have disease annotations (*i.e.*, those with both chemical and disease annotations).

The exclusion list, which contained 262 compounds, was generated by taking the union of the two lists of compounds identified with the above criteria. The two lists had 138 compounds in common, while 112 and 12 compounds were unique to the first and second lists, respectively. The exclusion list is available as supplementary information (see Additional File 1). Applying the exclusion list is not limited to filtering the results. The values of ${N}_{s,p}^{\left(t\right)}$ decrease on some occasions when excluded compounds are not counted. As a result of this, the values of ${\alpha }_{s,p}^{\left(t\right)}$ increase.

### Availability of data and source codes

For archival purposes, the co-occurrence dataset and analysis source codes used in this study were made available at Zenodo (10.5281/zenodo.15644308) [30]. The latest version of the patent co-occurrence data is freely accessible via PubChem (https://pubchem.ncbi.nlm.nih.gov). Users can explore the data interactively through the summary pages of individual compound and gene records or download it programmatically.

## Results

### Downloaded dataset used for analysis

We used a subset of the Google Patents Research Data (GPRD) dataset from October 2023, downloaded using Google BigQuery. Granted patents from the US, European, Japanese, and Korean Patent Offices have been included in the subset, resulting in a total of 24.40 million patent documents belonging to 18.99 million patent families. The entities annotated in the patent documents with the proprietary confidence scores equal to or greater than 0.4 were matched to records in PubChem, as explained in the Methods section. Statistics for annotations and database matching are shown in Tables 3 and 4. Note that only chemical annotations that have InChIKeys have been included. On the other hand, disease, gene, and protein annotations are matched by name.

**Table 3:** Statistics of chemical, disease, and gene-protein entities in granted patents from US, European, Japanese, and Korean Patent Offices with Google/OntoChem confidence score ≥ 0.4

Type	Number of patents with annotated entities	Number of patents with annotated entities matched to PubChem records	Percentage
Chemical	7.80 M	7.79 M	99.9%
Disease	2.24 M	1.54 M	68.8%
Genes and proteins	1.52 M	763.53 K	50.2%
TOTAL	8.57 M	8.23 M	96.0%

**Table 4:** Statistics of matched database identifiers for chemical, disease, and gene-protein entities in granted patents from US, European, Japanese, and Korean Patent Offices with Google/OntoChem confidence score ≥ 0.4

Type	Number of database identifiers	Number of database identifiers with at least one positive informativeness weight	Percentage
Chemical, matched, no. CIDs	10.45 M	5.92 M	56.7%
Disease, matched, no. MeSH IDs	7.24 K	6.43 K	88.8%
Genes and proteins, matched no. gene symbols, protein accessions or enzyme numbers	30.89 K	26.68 K	86.4%
TOTAL	10.49 M	5.95 M	56.7%

As shown in Table 4, 10.45 million PubChem compounds are matched to annotations in the patent documents considered in this study (with 5.92 million of them having a non-zero informativeness weight in at least one patent section and considered in the co-occurrence analysis). There are 7.24 thousand diseases and 30.90 thousand genes/proteins that are matched with annotations in the patent documents (with 6.43 thousand diseases and 26.68 thousand genes/proteins having a positive informativeness weight in at least one patent section and considered in the co-occurrence analysis). Overall, out of 10.49 million matched unique chemical, disease, and protein/gene database identifiers, 5.95 million (56.7%) have a positive informativeness weight in at least one patent section and are considered in the co-occurrence analysis.

Table 5 shows the most commonly occurring entities in this patent data set, along with the number of patent documents where the entities appeared. The most commonly occurring chemicals are CID 962 (water) and the effectively equivalent CID 22247451 (both in 3.83 million patents), followed by CID 5359268 (aluminum) (found in 1.51 million patents). The most common gene was Endogenous Retrovirus Group K Member 25 (ERVK-25) (92.33 thousand patents), followed by Insulin (INS) (87.33 thousand patents). The most common disease term mentioned was MeSH ID D009369 (Neoplasms) (284.69 thousand patents), followed by MeSH ID D007249 (Inflammation) (162.33 thousand patents).

**Table 5:** The top-10 most commonly occurring entities in the Google Patent data set considered in this study, along with the number of patent documents where they appeared (N_doc_). “Genes” include proteins and enzymes

	Chemicals		Diseases		Genes	
	Name	N_doc_	Name	N_doc_	Name	N_doc_
1	Water	3.83 M	Neoplasms	284.69 K	endogenous retrovirus group k member 25	92.33 K
2	Hydron;hydroxide	3.83 M	Inflammation	162.33 K	insulin	87.36 K
3	Aluminum	1.51 M	Hemorrhage	141.45 K	tubulin polymerization promoting protein	53.74 K
4	Carbon	1.49 M	Diabetes Mellitus	133.04 K	eukaryotic translation termination factor 1	49.02 K
5	Silicon Dioxide	1.37 M	Hypersensitivity	90.23 K	mitochondrial translation release factor 1	48.86 K
6	Ethanol	1.13 M	Breast Neoplasms	84.25 K	dna polymerase beta	48.84 K
7	Copper	1.12 M	Hypertension	78.28 K	renal disease susceptibility qtl 1	48.83 K
8	Nitrogen	1.11 M	Arteriosclerosis	75.10 K	peroxisome proliferator activated receptor delta	39.29 K
9	Oxygen	1.09 M	Pain	72.82 K	dna-directed rna polymerase i subunit rpa1	37.80 K
10	Iron	978.35 K	Asthma	71.21 K	galactosidase beta 1	36.34 K

### Inverse dataset frequency weights

The effective counts ${\overline{N} }_{i}^{\left({t}_{1},{t}_{2}\right)}$ are defined by the formula (5) and inverse dataset frequency terms ${r}_{i}^{\left({t}_{1},{t}_{2}\right)}$ are calculated by (4). Effective counts ${\overline{N} }_{i}^{\left(\text{chemical},\text{ chemical}\right)}$ and inverse dataset frequency terms ${r}_{i}^{\left(\text{chemical},\text{chemical}\right)}$ for selected compounds in the chemical-chemical dataset (with and without application of the chemical exclusion list) are shown in Table 6. In the chemical-chemical dataset, ${N}_{DS}^{\left(\text{chemicsl},\text{ chemical}\right)}=6.27\text{M}$ without application of the exclusion list, and ${N}_{DS}^{\left(\text{chemicsl},\text{ chemical}\right)}=6.28\text{M}$ with the exclusion list applied. Chemicals that very commonly appear in the patent documents (without the exclusion list applied) have large values of ${\overline{N} }_{i}^{\left(\text{chemical},\text{chemical}\right)}$ (1.05 M for water, 214.21 K for silicon dioxide, and 205.99 K for oxygen), resulting in small ${r}_{i}^{\left(c\text{hemical},\text{chemical}\right)}$ values (0.114 for water, 0.216 for silicon dioxide, and 0.218 for oxygen). Ibuprofen has ${r}_{i}^{\left(c\text{hemical},\text{chemical}\right)}$ values 0.546 and 0.562, with and without application of the exclusion list, respectively.

**Table 6:** Effective counts and inverse dataset frequency weights for selected compounds in the chemical-chemical dataset (with and without application of the chemical exclusion list).

CID	Name	Without application of the exclusion list		With application of the exclusion list	
		${\overline{N} }_{i}^{\left(\text{chemical},\text{chemical}\right)}$	${r}_{i}^{\left(\text{chemical},\text{chemical}\right)}$	${\overline{N} }_{i}^{\left(\text{chemical},\text{chemical}\right)}$	${r}_{i}^{\left(\text{chemical},\text{chemical}\right)}$
962	Water	1,050,520	0.114	n/a	n/a
24261	Silicon Dioxide	214,210	0.216	n/a	n/a
977	Oxygen	205,991	0.218	n/a	n/a
5359,268	Aluminum	201,701	0.220	n/a	n/a
947	Nitrogen	156,801	0.236	n/a	n/a
783	Hydrogen	155,105	0.236	n/a	n/a
23978	Copper	154,137	0.237	n/a	n/a
702	Ethanol	149,193	0.239	n/a	n/a
887	Methanol	62,036.8	0.295	n/a	n/a
6325610	Melanin	2847.52	0.492	3417.68	0.480
2244	Aspirin	2465.32	0.501	3061.50	0.487
36314	Paclitaxel	2106.55	0.511	2492.36	0.500
5743	Dexamethasone	1718.14	0.524	2176.23	0.509
774	Histamine	1541.02	0.531	1911.39	0.517
5460033	Cisplatin	1363.79	0.539	1648.82	0.527
23940	Plutonium	1172.23	0.548	1343.76	0.540
3672	Ibuprofen	955.644	0.562	1220.94	0.546
33613	Amoxicillin	402.192	0.617	486.832	0.605
4829	Pioglitazone	250.29	0.647	311.805	0.633
74164	N-Phenylmethacrylamide	18.4883	0.814	28.46	0.786
71312211	Laminaritetraose	4.29981	0.907	5.65	0.889
104888	( +)-alpha-Artemether	1.15705	0.991	1.75	0.964

Without restriction list: ${N}_{\text{DS}}^{(\text{chemical},\text{ chemical})}=6.267M$. With restriction list: ${N}_{\text{DS}}^{(\text{chemical},\text{ chemical})}=6.278M.$

### Using the exclusion list for chemical compound neighbors

As explained previously, patent documents often contain chemicals that are not the subject matter of the inventions. Such chemicals are not appropriate for inclusion in neighbor lists. Although their importance can be lessened by the use of IDF weight, this method is not always sufficient. To address this, an exclusion list was created (see above). The effects of filtering using the exclusion list, including how it adjusts the informativeness weights due to the application of the exclusion list, are illustrated below.

Tables 7, 8, 9 show the top-10, non-redundant neighbors for chemical, disease, and gene for chemotherapeutic agent CID 36314 (Paclitaxel), respectively. Tables 10 and 11 list top-10, non-redundant chemical neighbors for MeSH ID D009369 (Neoplasms) and Insulin, respectively. These five Tables show the effects of applying the exclusion list upon the order of the neighbors and their co-occurrence scores. While some differences are observed in the results with and without application of the exclusion list for chemical neighbors in Tables 7, 8, 9, the differences are very prominent in Tables 10 and 11.

**Table 7:** Top-10 non-redundant chemical neighbors for CID 36314 (Paclitaxel) with and without applying the exclusion list for chemical neighbors

	Without application of the exclusion list			With application of the exclusion list		
	CID	Name	Co-occurrence score	CID	Name	Co-occurrence score
1	148124	Docetaxel	228.84	148124	Docetaxel	265.90
2	31703	Doxorubicin	168.43	31703	Doxorubicin	202.83
3	5460033	Cisplatin	143.96	5460033	Cisplatin	169.42
4	5284616	Sirolimus	108.95	5284616	Sirolimus	128.67
5	3385	Fluorouracil	91.81	3385	Fluorouracil	110.43
6	10339178	Carboplatin	85.34	10339178	Carboplatin	99.23
7	60750	Gemcitabine	80.89	60750	Gemcitabine	91.91
8	5978	Vincristine	66.67	5978	Vincristine	81.55
9	36462	Etoposide	65.02	36462	Etoposide	78.36
10	702	Ethanol	64.94	30323	Daunorubicin	76.97

**Table 8:** Top-10 disease neighbors for CID 36314 (Paclitaxel) with and without applying the exclusion list for chemical neighbors

	Without application of the exclusion list			With application of the exclusion list		
	MeSH ID	Name	Co-occurrence score	MeSH ID	Name	Co-occurrence score
1	D009369	Neoplasms	310.71	D009369	Neoplasms	350.27
2	D001943	Breast Neoplasms	168.16	D001943	Breast Neoplasms	186.61
3	D010051	Ovarian Neoplasms	127.39	D010051	Ovarian Neoplasms	140.55
4	D008175	Lung Neoplasms	113.98	D008175	Lung Neoplasms	127.61
5	D003110	Colonic Neoplasms	89.73	D003110	Colonic Neoplasms	101.88
6	D011471	Prostatic Neoplasms	89.42	D011471	Prostatic Neoplasms	101.35
7	D003251	Constriction, Pathologic	83.96	D009362	Neoplasm Metastasis	94.23
8	D009362	Neoplasm Metastasis	83.57	D003251	Constriction, Pathologic	94.09
9	C562463	Pancreatic Carcinoma	77.93	C562463	Pancreatic Carcinoma	87.04
10	D010190	Pancreatic Neoplasms	76.67	D008545	Melanoma	86.59

**Table 9:** Top-10 gene neighbors for CID 36314 (Paclitaxel) with and without applying the exclusion list for chemical neighbors. Case-insensitive gene symbols are used in PubChem

	Without application of the exclusion list			With application of the exclusion list		
	Gene symbol	Name	Co-occurrence score	Gene symbol	Name	Co-occurrence score
1	TUBB4B	tubulin	47.18	TUBB4B	tubulin	52.83
2	ASPG	asparaginase	39.24	VEGFA	vascular endothelial growth factor a	44.45
3	VEGFA	vascular endothelial growth factor a	38.71	ASPG	asparaginase	43.10
4	ERBB2	erb-b2 receptor tyrosine kinase 2	31.83	ERBB2	erb-b2 receptor tyrosine kinase 2	35.58
5	CSF2	colony stimulating factor 2	26.84	CSF2	colony stimulating factor 2	30.41
6	EGF	epidermal growth factor	25.53	INS	insulin	30.02
7	INS	insulin	24.84	EGF	epidermal growth factor	29.49
8	EGFR	epidermal growth factor receptor	23.25	TPPP	tubulin polymerization promoting protein	26.99
9	TPPP	tubulin polymerization promoting protein	22.96	EGFR	epidermal growth factor receptor	26.21
10	ERVK-25	endogenous retrovirus group K member 25	22.58	ERVK-25	endogenous retrovirus group K member 25	26.21

**Table 10:** Top-10 non-redundant chemical neighbors for MeSH ID D009369 (Neoplasms) with and without applying the exclusion list for chemical neighbors

	Without application of the exclusion list			With application of the exclusion list		
	CID	Name	Co-occurrence score	CID	Name	Co-occurrence score
1	962	Water	1992.74	31703	Doxorubicin	717.22
2	702	Ethanol	1339.01	36314	Paclitaxel	687.06
3	5234	Sodium Chloride	1220.79	5460033	Cisplatin	582.95
4	516892	Sodium Bicarbonate	1167.49	5904	Penicillin G	516.41
5	783	Hydrogen	1145.65	19649	Streptomycin	510.98
6	947	Nitrogen	967.11	6106	Leucine	492.46
7	977	Oxygen	858.86	18730	Fluorescein-5-isothiocyanate	480.54
8	6547	2,2'-Azobis(2-methylpropionitrile)	830.43	5950	Alanine	461.61
9	5793	D-Glucose	776.59	6274	Histidine	457.98
10	5462310	Carbon	755.45	6137	Methionine	454.62

**Table 11:** Top-10 non-redundant chemical neighbors for gene symbol INS (insulin) with and without applying the exclusion list for chemical neighbors

	Without application of the exclusion list			With application of the exclusion list		
	CID	Name	Co-occurrence score	CID	Name	Co-occurrence score
1	962	Water	1001.14	16132283	Glucagon	274.39
2	702	Ethanol	584.93	22833565	Sodium heparin	176.45
3	516892	Sodium Bicarbonate	513.71	16133831	Glucagon-like peptide-I(7–36) amide	173.35
4	5234	Sodium Chloride	484.89	4091	Metformin	155.99
5	5793	D-Glucose	351.41	23663392	Hyaluronic acid sodium salt	145.57
6	947	Nitrogen	349.71	16157882	Exenatide free base	145.40
7	6049	Edetic Acid	312.26	5743	Dexamethasone	135.78
8	753	Glycerin	308.44	5754	Hydrocortisone	133.75
9	6547	2,2'-Azobis(2-methylpropionitrile)	303.63	5904	Penicillin G	132.09
10	5462310	Carbon	293.01	16129706	Somatostatin	131.46

There is a substantial overlap between the top-10 chemical neighbors lists for neoplasms (Table 10) and insulin (Table 11), with eight chemicals appearing in both lists. Notably, all top-10 chemical neighbors for these two entities are included in the exclusion list. Therefore, the application of the exclusion list removes all these chemicals, resulting in completely different sets of top-10 chemical neighbors for the query entities. The resulting neighbor lists are more specific to the queries, containing known anti-cancer drugs (e.g., doxorubicin, paclitaxel, and cisplatin) and anti-diabetic drugs (e.g., metformin and exenatide). However, applying the exclusion list can lead to the loss of a well-known entity relationship, as exemplified with D-glucose removed from the neighbor list for insulin.

It is worth mentioning that the exclusion list for chemical neighbors only affects neighbor relationships where chemical entities are involved either as the query entity Tables 7, 8, 9 or neighbors (Tables 7, 10, and 11). It does not affect the neighbor relationships that do not involve chemicals (e.g., gene–gene, gene-disease, disease-gene, and disease-disease relationships).

### Implementation of the patent knowledge panels

**Fig. 4:**
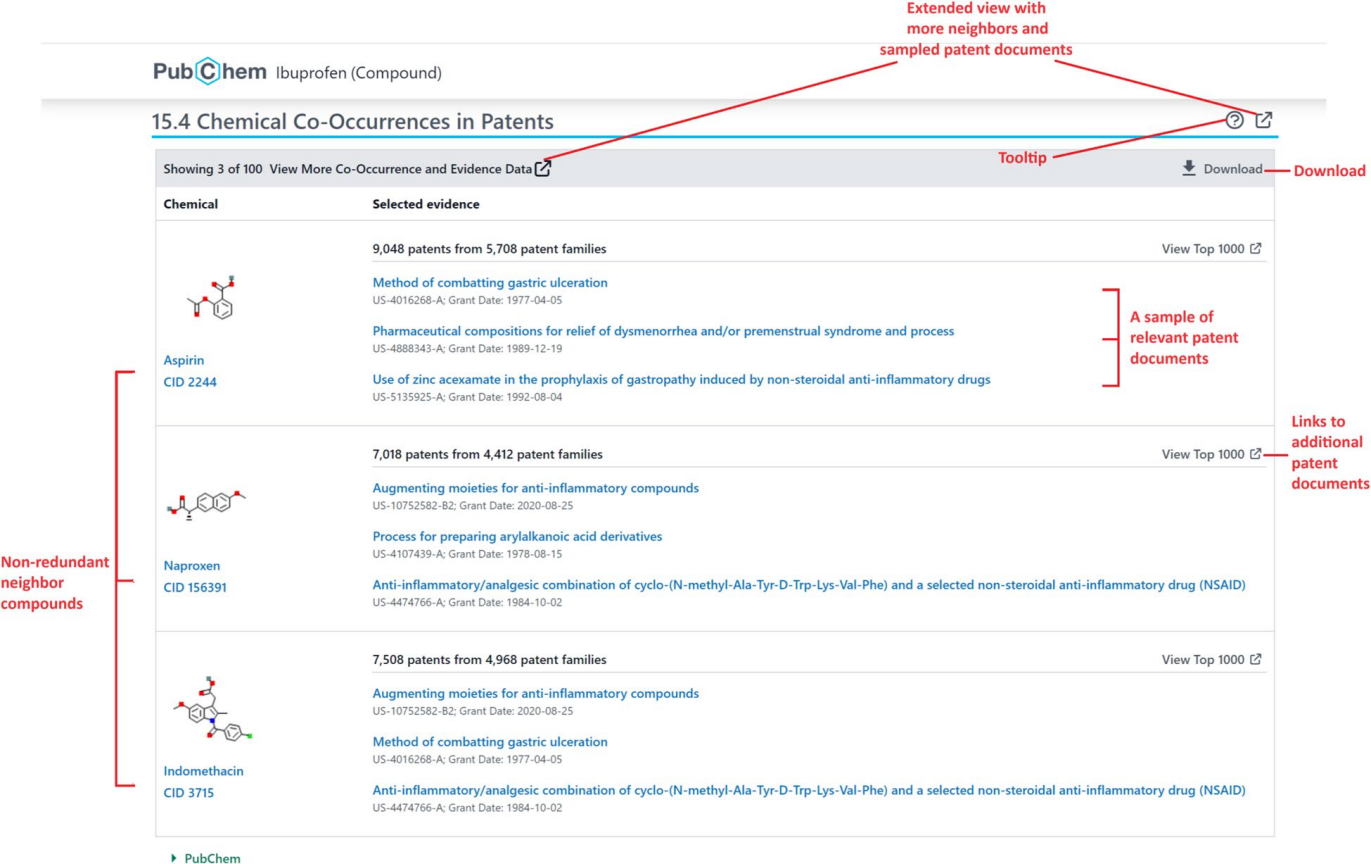
The “Chemical Co-Occurrences in Patents” section of the PubChem Compound summary page for ibuprofen (CID 3672) (https://pubchem.ncbi.nlm.nih.gov/compound/3672#section=Chemical-Co-Occurrences-in-Patents)

The design of the co-occurrence panels for patents is very similar to that for literature described in our previous paper [8]. The precalculated co-occurrence data is loaded into a set of databases and used to generate the patent knowledge panels presented in the Patents section of the summary pages for PubChem Compound and Gene records (PubChem does not provide disease-related pages). The links to sample patent co-occurrent panels are listed in Table 12. A screenshot of the chemical-chemical co-occurrence panel for ibuprofen (CID 3672) is shown in Fig. 4. By default, the patent knowledge panel shows the top-3 neighbor records co-occurring in patent documents with the query record, along with the three most relevant patent families for each neighbor. These relevant patent families are selected using the relevance score. Clicking the “View More” button opens an extended knowledge panel view that shows more neighbors and relevant patent documents. The “View Top 1000” button allows users to get a list of up to 1000 relevant patent documents for a given query-neighbor pair. The data presented in the knowledge panel can be downloaded in various formats using the pop-up selection menu.

**Table 12:** Links to the patent knowledge panels for CID 36314 (paclitaxel) and for the EGFR gene

Co-occurrence type	Link
Chemical-chemical	https://pubchem.ncbi.nlm.nih.gov/compound/36314#section=Chemical-Co-Occurrences-in-Patents
Chemical gene	https://pubchem.ncbi.nlm.nih.gov/compound/36314#section=Chemical-Gene-Co-Occurrences-in-Patents
Chemical-disease	https://pubchem.ncbi.nlm.nih.gov/compound/36314#section=Chemical-Disease-Co-Occurrences-in-Patents
Gene-chemical	https://pubchem.ncbi.nlm.nih.gov/gene/egfr#section=Gene-Chemical-Co-Occurrences-in-Patents
Gene–gene	https://pubchem.ncbi.nlm.nih.gov/gene/egfr#section=Gene-Gene-Co-Occurrences-in-Patents
Gene-disease	https://pubchem.ncbi.nlm.nih.gov/gene/egfr#section=Gene-Disease-Co-Occurrences-in-Patents

### Programmatic interfaces

The patent co-occurrence data in PubChem can be accessed programmatically using the panels’ underlying web (HTTP) service. The syntax of a programmatic access request is:

https://pubchem.ncbi.nlm.nih.gov/link_db/link_db_server.cgi?format=ARG1&type=ARG2&operation=ARG3&id_1=ARG4&response_type=ARG5

where the possible parameter values (values of ARG1 through ARG5) are listed in Table 13. For example, the following URL allows the user to get a list of chemicals mentioned with CID 3672 in patent documents in JSON:

**Table 13:** Parameters for the PubChem interface for co-occurrence neighbors (https://pubchem.ncbi.nlm.nih.gov/link_db/link_db_server.cgi)

Parameter	Values	Is Optional ^*a*^
Type	PatentChemicalChemical PatentChemicalDisease PatentChemicalGene PatentGeneChemical PatentGeneDisease PatentGeneGene PatentDiseaseChemical PatentDiseaseDisease PatentDiseaseGene	No
Format	**XML** JSON ASNT	Yes
Operation	GetAllLinks	No
id_1 (query ID)	< CID > < disease MeSH ID > < gene_symbol >	No
response_type	**display** save	Yes

^*a*^When the optional parameters are omitted, its default value (indicated in bold) will be used

https://pubchem.ncbi.nlm.nih.gov/link_db/link_db_server.cgi?format=JSON&type=PatentChemicalChemical&operation=GetAllLinks&id_1=3672

In this example, the “response_type” parameter is omitted, and the default value “display” is used, which displays the requested co-occurrence data. The other possible value for this parameter is “save”, which downloads the data. Additional request URL examples for programmatic access to patent co-occurrence data are listed in Table 14.

**Table 14:** Examples of programmatic download queries

Co-occurrence type	An example of query
Chemical-chemical	https://pubchem.ncbi.nlm.nih.gov/link_db/link_db_server.cgi?format=JSON&type=PatentChemicalChemical&operation=GetAllLinks&id_1=3672
Chemical-disease	https://pubchem.ncbi.nlm.nih.gov/link_db/link_db_server.cgi?format=JSON&type=PatentChemicalDisease&operation=GetAllLinks&id_1=3672
Chemical-gene	https://pubchem.ncbi.nlm.nih.gov/link_db/link_db_server.cgi?format=JSON&type=PatentChemicalGene&operation=GetAllLinks&id_1=3672
Gene-chemical	https://pubchem.ncbi.nlm.nih.gov/link_db/link_db_server.cgi?format=JSON&type=PatentGeneChemical&operation=GetAllLinks&id_1=ptgs2
Gene–gene	https://pubchem.ncbi.nlm.nih.gov/link_db/link_db_server.cgi?format=JSON&type=PatentGeneGene&operation=GetAllLinks&id_1=ptgs2&response_type=display
Gene-disease	https://pubchem.ncbi.nlm.nih.gov/link_db/link_db_server.cgi?format=JSON&type=PatentGeneDisease&operation=GetAllLinks&id_1=ptgs2
Disease-chemical	https://pubchem.ncbi.nlm.nih.gov/link_db/link_db_server.cgi?format=JSON&type=PatentDiseaseChemical&operation=GetAllLinks&id_1=D013927
Disease-gene	https://pubchem.ncbi.nlm.nih.gov/link_db/link_db_server.cgi?format=JSON&type=PatentDiseaseGene&operation=GetAllLinks&id_1=D013927
Disease-disease	https://pubchem.ncbi.nlm.nih.gov/link_db/link_db_server.cgi?format=JSON&type=PatentDiseaseDisease&operation=GetAllLinks&id_1=D013927

## Discussion

### Comparison of co-occurrence data derived from patents vs. PubMed

The co-occurrence data used in the literature knowledge panels is extracted by the PubChem team from PubMed records with text mining software [20], as described in our previous study [8]. Because PubMed records do not contain full text, the entities are extracted only from the articles’ titles and abstracts. On the other hand, the co-occurrence data used in the patent knowledge panel is derived by mapping PubChem record identifiers with annotations from the GPRD set, which come not only from the title and abstract of a patent document but also from other parts, including claims and descriptions. Although we could have used only the entities appearing in the title and abstract of a patent, it was necessary in this study to include entities from other parts of the patent because patent documents are different from scientific articles in significant ways. Patents are legal documents written in a very specialized language and format. Also, because titles and abstracts in patents are required to be short [31, 32], entities directly related to the subject matter of the invention often appear in the claims and description sections, which can vary in length significantly between patent documents.

Table 15 shows the total number of entity relationships for each of the query-neighbor types. There are more chemical-chemical, chemical-gene, and chemical-disease pairs from patents than those from PubMed records, implying that the coverage of chemical entities in the patent knowledge panels is much broader than in the literature knowledge panels based on PubMed records. This may be because the GPRD set covers knowledge areas beyond the life and biomedical sciences (e.g., material sciences, nanotechnology, and industrial chemistry) and because we considered entities from other sections beyond the patent title and abstract. In contrast, for gene and disease queries, patent documents resulted in fewer query-neighbor pairs than PubMed records (e.g., 26 K vs. 41 K gene-chemical pairs for patents and PubMed records, respectively, and 6 K vs. 8 K disease-chemical pairs for patents and PubMed records, respectively). This reflects the fact that patent documents typically focus on genes and diseases that are deemed to be good targets for commercialization purposes, while scientific articles deal with a broader set of genes and diseases.

**Table 15:** Number of query DB IDs in different co-occurrence datasets in patent records and literature (PubMed) records

Co-occurrence type	Number of query DB IDs for co-occurrences in patents (with application of the exclusion list)	Number of query DB IDs for co-occurrences in PubMed records
Chemical-chemical	6.146 M	283.609 K
Chemical-disease	3.615 M	169.144 K
Chemical-gene	2.955 M	169.983 K
Gene-chemical	26.485 K	41.420 K
Gene-disease	25.881 K	41.890 K
Gene–gene	26.617 K	44.949 K
Disease-chemical	6.343 K	8.036 K
Disease-disease	6.413 K	9.060 K
Disease-gene	6.258 K	8.298 K

Tables 16, 17, and 18 compare the top-10 chemical, gene, and disease neighbors of ethanol (CID 702) from PubMed records with those from patent documents. As shown in Table 16, only one chemical neighbor (acetaldehyde (CID 177)) appears in both lists, implying that PubMed records and patent documents have very different chemical coverages. The observed difference between the two lists is in part due to the use of the exclusion list to suppress chemicals that are not likely to be the subject matter of patents. For example, while water (CID 962) and D-glucose (CID 5793) appear in the neighbor list from PubMed records, they do not appear in the list from patents. It is noteworthy that five of the chemical neighbors from patents (isobutanol, 1-pentanol, 1-hexanol, 1-octanol, and cetyl alcohol) are monohydric alcohols (i.e., alcohols with one hydroxyl group), whereas the neighbor list from PubMed records has only one monohydric alcohol (methanol).

**Table 16:** Top-10 chemical neighbors of ethanol (CID 702) in PubMed records and patent documents

	PubMed records		Patents	
	CID	Chemical Name	CID	Chemical Name
1	962	Water	6912	Xylitol
2	5793	D-Glucose	6560	Isobutanol
3	887	Methanol	169019	D-Threitol
4	177	Acetaldehyde	177	Acetaldehyde
5	446220	Cocaine	6276	1-Pentanol
6	5997	Cholesterol	23663392	Hyaluronic acid sodium salt
7	5988	Sucrose	493591	Maltitol
8	180	Acetone	957	1-Octanol
9	5462310	Carbon	2682	Cetyl Alcohol
10	89,594	Nicotine	8103	1-Hexanol

**Table 17:** Top-10 gene neighbors of ethanol (CID 702) in PubMed records and patent documents

	PubMed records		Patents	
	Gene Identifier	Gene Name	Gene Identifier	Gene Name
1	EC:1.1.1.1	Alcohol dehydrogenase	EC:3.2.1.4	Cellulase
2	GPT	Glutamic-pyruvic transaminase	ERVK-25	Endogenous retrovirus group K member 25
3	INS	Insulin	LIPE	Lipase E
4	CAT	Catalase	EC:1.14.18.1	Tyrosinase
5	EC:2.6.1.1	Aspartate transaminase	MGAM	Maltase-glucoamylase
6	EC:2.3.2.2	γ-Glutamyltransferase	EC:1.1.1.1	Alcohol dehydrogenase
7	CYP2E1	Cytochrome P450 2E1	EC:3.2.1.3	Glucan 1,4-α-glucosidase
8	ALDH2	Aldehyde dehydrogenase 2	INS	Insulin
9	EC:1.15.1.1	Superoxide dismutase	ELN	Elastin
10	IL6	Interleukin 6	EC:3.2.1.15	Endo-polygalacturonase

**Table 18:** Top-10 disease neighbors of ethanol (CID 702) in PubMed records and patent documents

	PubMed records		Patents	
	MeSH ID	Disease name	MeSH ID	Disease name
1	D000437	Alcoholism	D009369	Neoplasms
2	D019966	Substance-related disorders	D007249	Inflammation
3	D001523	Mental disorders	D003920	Diabetes mellitus
4	D003866	Depressive disorder	D012871	Skin diseases
5	D000435	Alcoholic intoxication	D006967	Hypersensitivity
6	D001008	Anxiety disorders	D006973	Hypertension
7	D008107	Liver diseases	D009765	Obesity
8	D003920	Diabetes mellitus	D003876	Dermatitis, atopic
9	D009369	Neoplasms	D006470	Hemorrhage
10	D063647	Fetal alcohol spectrum disorders	D001161	Arteriosclerosis

Among ethanol’s top-10 gene neighbors from PubMed records and patents (Table 17), only two genes (alcohol dehydrogenase and insulin) are common in both. Similarly, only two diseases (neoplasms and diabetes mellitus) appeared in both lists of disease neighbors of ethanol (Table 18). The data in Tables 16, 17, 18 indicates that PubMed records and patents have very different coverage of chemicals, genes, and diseases.

### Limitations and future direction

Two entities are co-mentioned in a patent document for various reasons. As an example, Table 19 shows top-10 disease neighbors for MeSH ID D009369 (Neoplasms), along with their co-occurrence score. Many of these neighbors are types of neoplasms that occur in different organs (*e.g.*, breast, lung, prostatic, colonic, and ovarian neoplasms). Similarly, melanoma, leukemia, and carcinoma are malignant (cancerous) neoplasms. On the other hand, MeSH ID D007249 (Inflammation) is also listed among the top-10 neighbors. Inflammation and cancer are closely related, and chronic inflammation is considered one of the key factors that can contribute to the development and progression of cancer [33]. One of the limitations of the co-occurrence data derived from patents (as well as those derived from PubMed records in our previous study [8]) is that it does not capture the context in which two entities are co-mentioned in a patent (that is, why they commonly appear together in a patent). To definitively learn this, one would need to read the patent. However, this limitation is addressed in part by providing a sample of relevant patent documents co-mentioning the entities, as implemented in the patent knowledge panel (Fig. 4).

**Table 19:** Top-10 disease neighbors for MeSH ID D009369 (Neoplasms) in patent documents

	MeSH ID	Name	Co-occurrence score
1	D001943	Breast Neoplasms	2614.62
2	D009362	Neoplasm Metastasis	1881.75
3	D008175	Lung Neoplasms	1802.55
4	D011471	Prostatic Neoplasms	1719.05
5	D003110	Colonic Neoplasms	1640.39
6	D007249	Inflammation	1544.99
7	D008545	Melanoma	1535.65
8	D007938	Leukemia	1499.11
9	D002277	Carcinoma	1452.48
10	D010051	Ovarian Neoplasms	1368.52

The annotations provided with the Google Patent Research Data (GPRD) do not allow one to distinguish entities directly related to the novel claims of a patent from those mentioned for other reasons (and the authors are not aware of any current annotation technology providing a deep understanding like this). In the present study, this issue was not explicitly addressed; however, the use of the informativeness weights, the inverse dataset frequency weights, and the exclusion list helps mitigate this issue in part.

It is difficult, if not impossible, to extract using co-occurrence analysis any useful information about entities that are only mentioned in a patent section along with many other entities of the same type. As a result, only a subset of GPRD’s annotated entities is present in this analysis, and the informativeness of the results for any given entity may vary.

Another limitation is that the GPRD set is from automatic translations of non-English patents (*e.g.*, those from the Japan Patent Office (JPO) and the Korean Intellectual Property Office (KIPO)) by an algorithm developed and utilized by Google, which is not known to us. In this study, the granted patents from four different jurisdictions were considered: the US (USPTO), Europe (EPO), Japan (JPO), and Korea (KIPO). The latter three patent agencies are known to be reasonably well-aligned in patent corpuses with the USPTO. For patents not well-represented by these agencies, extension of this work to other jurisdictions may be hampered by poor automatic translations.

The informativeness weights are empirically determined. They would be expected to be better than constant weights, but we do not know if they are optimal. Despite all our efforts to utilize informativeness weights and inverse dataset frequencies, it seemed necessary to employ a small exclusion list to remove some commonly occurring compounds. However, it should be emphasized that whether or not to add a chemical to the exclusion list is a very subjective decision. While the chemicals on the current exclusion list were selected by the PubChem team, the list may be updated in the future based on feedback from PubChem users.

Under an abundance of caution, we prioritized correctness over novelty, and considered only granted patents from the four patent agencies listed above. The benefits and dangers of including patent applications that are not yet granted is an interesting issue that could be studied separately.

In this study, only chemical entities annotated in GPRD with InChIKeys (with assigned types *chemCompound*, *drugs*, *inorgmat*, *natprod*, *polymers*, and *substances*) were matched to PubChem compounds. The entities of the listed types without InChI keys were ignored, including those with chemical names only. There is an ongoing project within PubChem to supplement PubChem compounds with names from various other authoritative and curated sources, which could in the future be used to match to GPRD’s chemical names.

## Conclusions

In this study, we present an algorithm for the co-occurrence analysis of chemicals, genes, and diseases in patent documents annotated by the GPRD set. The resulting data is used to generate the patent knowledge panels in PubChem pages, which allow users to quickly identify entities commonly mentioned in patents alongside a given chemical or gene. The data available through the patent co-occurrence panels can be downloaded, enabling further exploration and analysis; the data can also be accessed programmatically. Although there is always room for improvement, we expect the patent knowledge panels and the underlying data to be highly valuable to PubChem users to rapidly identify key correlations between chemicals, genes, and diseases that may not be readily discoverable by other means.

## Supplementary Information

Supplementary file 1. The exclusion list containing 262 chemicals generated in this study.

## Data Availability

The latest version of the patent co-occurrence data is freely available at PubChem (https://pubchem.ncbi.nlm.nih.gov). They can be accessed interactively through the summary pages of individual compound and gene records and downloaded programmatically. For archival purposes, the co-occurrence data used in this study and the source code for analysis are freely available at Zenodo (10.5281/zenodo.15644308) [30].

## Abbreviations

EPO: European Patent Office
GPRD: Google Patents Research Data
InChI: The International Chemical Identifier
JPO: Japan Patent Office
KIPO: Korean Intellectual Property Office
NCBI: National Center for Biotechnology Information
NLP: Natural language processing
USPTO: United States Patent and Trademark Office
